# Effect of Metal Ions on the Conductivity, Self-Healing, and Mechanical Properties of Alginate/Polyacrylamide Hydrogels

**DOI:** 10.3390/ma18163871

**Published:** 2025-08-18

**Authors:** Chen-Kang Chen, Chien-Yin Lin, Rajan Deepan Chakravarthy, Yu-Hsu Chen, Chieh-Yi Chen, Hsin-Chieh Lin, Mei-Yu Yeh

**Affiliations:** 1 Department of Chemistry, Chung Yuan Christian University, Taoyuan 320314, Taiwan; za14253698a@gmail.com (C.-K.C.); lin.sylvia99999@gmail.com (C.-Y.L.); 2 Department of Materials Science and Engineering, National Yang Ming Chiao Tung University, Hsinchu 300093, Taiwan; deepannycu@gmail.com; 3 Department of Orthopedic Surgery, Taoyuan General Hospital, Ministry of Health and Welfare, Taoyuan 330215, Taiwan; magister.yuhsu@gmail.com; 4 Neurosurgical Department, Taoyuan General Hospital, Ministry of Health and Welfare, Taoyuan 330215, Taiwan; 5 Center for Intelligent Drug Systems and Smart Bio-Devices (IDS^2^B), National Yang Ming Chiao Tung University, Hsinchu 30068, Taiwan

**Keywords:** conductive hydrogels, alginate, polyacrylamide, metal ions, self-healing

## Abstract

Conductive hydrogels hold great promise for biomedical and electronic applications. However, their practical use is often limited by poor self-healing capability, which can affect long-term stability and durability. To address this, we developed alginate/polyacrylamide-based conductive hydrogels incorporating FeCl_3_ and AlCl_3_, named CH-Fe and CH-Al, respectively. We systematically studied the influence of metal cations on the hydrogels’ mechanical and electrical properties. CH-Al showed the most optimized performance, with a 329% increase in tensile strength and a 323% improvement in conductivity compared to the blank hydrogel. Additionally, CH-Al demonstrated excellent self-healing ability, with nearly 100% recovery after damage. The introduction of Al^3+^ improved conductivity by forming dynamic electron-conductive pathways through interactions with the polymer network. The self-healing behavior arises from reversible metal–ligand coordination bonds, which enable rapid recovery of the hydrogel’s structure after mechanical disruption. This study successfully developed a conductive hydrogel that combines high electrical conductivity, robust mechanical strength, and an intrinsic self-healing ability, offering significant potential for applications in bioelectronic devices, flexible sensors, and implantable medical technologies.

## 1. Introduction

Hydrogels are soft materials with a three-dimensional network structure that can absorb large amounts of water without dissolving [1,2,3]. They exhibit excellent biocompatibility and tunable mechanical properties, making them highly attractive for applications in flexible electronics [4,5], biomedical materials [6,7], tissue engineering [7,8,9], and drug delivery [10,11,12]. However, conventional hydrogels often suffer from limitations such as a lack of self-healing ability and poor electrical conductivity, which restrict their use in high-performance applications like wearable devices, smart sensors, and soft robotics. To overcome these challenges and expand their functional scope, recent research has focused on developing multifunctional composite hydrogels [13,14,15,16,17]. By introducing design strategies such as dynamic bonding, self-assembly, and conductive fillers, researchers aim to create advanced materials that combine mechanical robustness, self-healing capability, and electrical conductivity. In particular, conductive hydrogels have attracted increasing attention due to their ability to simultaneously conduct electrical signals and maintain high water content, offering promising prospects for bioelectronics and soft machines. Recent advances have explored the incorporation of various conductive components such as conductive polymers (e.g., polyaniline and PEDOT/PSS), metal ions, and carbon-based materials (e.g., carbon nanotubes and graphene). These components enhance the electrical conductivity of hydrogels while preserving their inherent softness and biocompatibility, thereby enabling their use in wearable biosensors, neural interfaces, and tissue-stimulating scaffolds [4,5].

Sodium alginate, a natural polysaccharide extracted from brown seaweed, forms hydrogels through ionic crosslinking when exposed to multivalent cations [18,19,20]. These hydrogels offer excellent biocompatibility, biodegradability, water retention, and mild gelation conditions, making them valuable in biomedical and environmental applications [21,22,23,24]. In biomedicine, alginate hydrogels are used for tissue engineering scaffolds, cell encapsulation, drug delivery, and wound dressings due to their soft, moisture-retaining properties that support healing [25,26,27,28]. They are also widely applied in the food industry as thickeners, gelling agents, or encapsulation systems to control flavor and nutrient release [29,30]. In agriculture, they serve in slow-release fertilizers and pesticides to enhance efficiency and reduce environmental impact [31,32,33]. Owing to their biocompatibility, design flexibility, and renewable nature, alginate hydrogels offer significant potential across diverse applications. With the integration of advanced polymers or nanomaterials, their mechanical properties and functional versatility can be further enhanced, paving the way for innovative uses in smart biomedical devices and environmentally sustainable technologies.

Polyacrylamide (PAAM) hydrogel is a soft, water-rich polymer network formed through the free-radical polymerization of acrylamide monomers [34,35]. It offers excellent biocompatibility, low cytotoxicity, and tunable mechanical properties, making it highly suitable for biomedical applications [36]. The mechanical characteristics of PAAM hydrogels—including elasticity, stretchability, and toughness—can be precisely adjusted by modifying crosslinking density or monomer concentration, or introducing comonomers and reinforcing agents. Owing to their high water content and porous structure, PAAM hydrogels can effectively mimic the physical and biochemical environment of biological tissues, making them ideal for use in artificial skin, wound healing, drug delivery systems, and tissue engineering scaffolds [37,38,39,40,41,42]. Furthermore, the hydrogel’s structural versatility allows for functional enhancements through the incorporation of conductive fillers such as carbon nanotubes, graphene, or metal nanoparticles [43,44,45,46]. These modifications endow the hydrogel with electrical conductivity while preserving its inherent flexibility, enabling applications in wearable electronics, electronic skin, strain sensors, and other soft, responsive electronic devices.

In this study, we successfully developed a series of conductive hydrogels based on a composite network of alginate and polyacrylamide, incorporating multivalent metal ions to enhance both mechanical and electrical performance. Specifically, FeCl_3_ and AlCl_3_ were introduced as ionic crosslinkers, and the resulting hydrogels were designated as CH-Fe and CH-Al, respectively. These metal ions were chosen for their strong coordination abili-ties, which play a crucial role in forming dynamic, reversible networks that contribute to the material’s overall performance. Our primary objective was to investigate the influence of these metal cations on the structural, mechanical, electrical, and self-healing properties of the hydrogels. The CH-Al hydrogel developed in this study offers a viable solution, addressing common limitations of traditional hydrogels such as mechanical fragility and lack of conductivity. Future research may further explore the integration of other functional components or responsive mechanisms, expanding the hydrogel’s capabilities and opening new possibilities in smart materials and bio-integrated systems.

## 2. Materials and Methods

### 2.1. Materials

Acrylamide and polyacrylamide (MW ~10,000) were acquired from Acros Organics (Geel, Antwerp, Belgium). Sigma-Aldrich (St. Louis, MO, USA) supplied potassium persulfate and sodium alginate. *N*,*N*′-Methylenebisacrylamide, aluminum chloride, and iron(III) chloride hexahydrate were purchased from Alfa Aesar (Ward Hill, MA, USA). Methacrylated lysine (LysMA) was synthesized following the protocol described in our previous study [47]. All experiments were conducted using deionized water.

### 2.2. Composite Hydrogel (CH) Preparation Protocol

Acrylamide (400 mg), sodium alginate (50 mg), and LysMA (30 mg) were dissolved in 2.5 mL of deionized water to form a clear solution. Subsequently, 500 μL of potassium persulfate solution (0.02 g/mL), 500 μL of polyacrylamide solution (1 mg/mL), and 100 μL of *N*,*N*′-methylenebisacrylamide solution (1 mg/mL) were added and thoroughly mixed to yield a homogeneous precursor solution. The mixture was then incubated at 70 °C for 6 h to initiate polymerization. After polymerization, the resulting hydrogels underwent different post-treatment steps depending on the formulation. For the blank hydrogel, no further treatment was applied. For CH-Al, the hydrogel was cooled to room temperature and immersed in a 0.4 M AlCl_3_ aqueous solution for 6 h to allow ionic crosslinking. For CH-Fe, the hydrogel was similarly cooled to room temperature and soaked in a 0.4 M FeCl_3_ aqueous solution for 6 h to facilitate ionic crosslinking.

### 2.3. Measurements

Mechanical properties were evaluated using a universal testing machine (Gotech AI-3000-U, Taichung, Taiwan) at a strain rate of 20 mm/min. Lap-shear tests were performed to evaluate the adhesion strength of the hydrogels using a 10 N load cell at a crosshead speed of 10 mm/min. Rheological behavior of the composite hydrogel samples was assessed using a rotational rheometer (DHR-1, TA Instruments, New Castle, DE, USA) equipped with a parallel plate geometry (20 mm diameter, 1 mm gap). Dynamic oscillatory frequency sweep tests were performed at 25 °C with a constant strain of 1%, and a measuring storage modulus (G′) and loss modulus (G″) over an angular frequency range of 0.1–100 rad/s. Additionally, strain amplitude sweep tests (γ = 1–10,000%, ω = 1 rad/s) were conducted at 25 °C to evaluate viscoelastic properties. Alternating strain cycles were applied, switching from small strain (γ = 10%) to large strain (γ = 2000%), with each interval lasting 200 s.

The surface morphology of the hydrogels was examined using a scanning electron microscope (SEM, JSM-7600F, JEOL, Tokyo, Japan). For SEM sample preparation, hydrogels were rapidly frozen in liquid nitrogen and subsequently lyophilized under vacuum at −80 °C for at least 24 h to ensure complete sublimation of water. The resulting freeze-dried samples were coated with a thin layer of platinum under vacuum prior to imaging [48,49].

Electrochemical measurements were performed using a CHI627E Electrochemical Workstation (CH Instruments, Austin, TX, USA). A sandwich-structured electrochemical cell was assembled by placing a conductive hydrogel sheet between two indium tin oxide (ITO) glass substrates, with the hydrogel layer having a thickness of 0.08 mm. Electrical conductivity (σ, S/cm) was determined based on the method described in our previous study [47].

## 3. Results and Discussions

### 3.1. Fabrication of Composite Hydrogels

**Figure 1:**
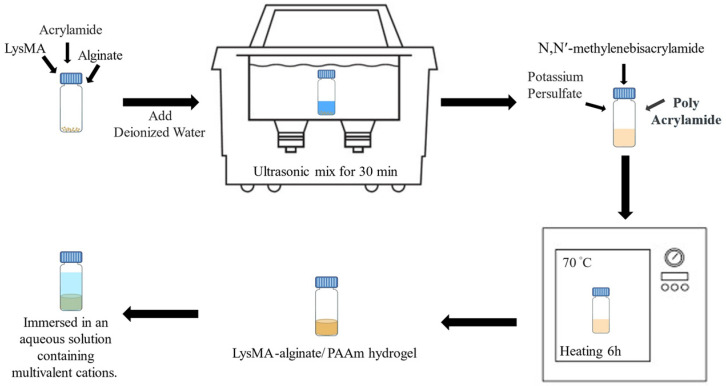
Approaches to the preparation of composite hydrogels.

**Scheme 1:**
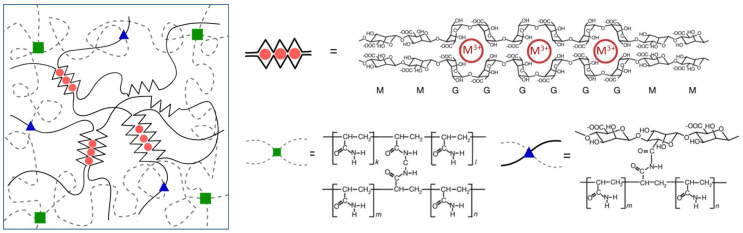
Proposed network structure of the composite hydrogel. M^3+^ represents trivalent cations such as Al^3+^ or Fe^3+^.

In this study, we selected two types of crosslinkable polymers—sodium alginate and acrylamide—to prepare hydrogels with enhanced mechanical properties. These polymers enable the formation of ionic and covalent crosslinking networks, respectively. Sodium alginate is a polysaccharide composed of β-D-mannuronic acid (M) and α-L-guluronic acid (G) units, arranged in block copolymers of M-M, G-G, or M-G sequences. In the presence of multivalent cations, the Na^+^ ions on the G blocks are exchanged with these cations, forming an ionically crosslinked network [50]. Acrylamide, on the other hand, undergoes polymerization initiated by potassium persulfate to form polyacrylamide (PAAM), resulting in a covalently crosslinked network. To further enhance the adhesive and mechanical properties of the hydrogel, *N*,*N*′-methylenebisacrylamide (MBAA) was incorporated as a crosslinker, increasing crosslink density and thus improving mechanical strength. Additionally, methacrylated lysine (LysMA) was introduced to copolymerize with acrylamide and impart adhesive functionality to the hydrogel. As illustrated in Figure 1, sodium alginate, acrylamide, and LysMA were first dissolved in deionized water to create a homogeneous precursor solution. Potassium persulfate was then added as an initiator for radical polymerization, followed by the addition of MBAA and PAAM. The addition of PAAM at this stage further improves the mechanical property of the hydrogel [49]. The mixture was heated at 70 °C for 6 h to form the hydrogel, referred to as the blank hydrogel. To further enhance mechanical strength through ionic crosslinking, multivalent cations (Al^3+^ and Fe^3+^) were introduced to crosslink the alginate chains. Using a straightforward two-step process, the blank hydrogel was immersed in aqueous solutions of AlCl_3_ and FeCl_3_ for 6 h to promote ion exchange and uniform ionic crosslinking. The resulting composite hydrogels were designated CH-Al and CH-Fe, respectively. Scheme 1 illustrates the proposed network structure of the composite hydrogel. Trivalent cations (Al^3+^ and Fe^3+^) serve as ionic crosslinkers by coordinating with the carboxylate and hydroxyl groups present on alginate chains, thereby forming a physically crosslinked ionic network. Meanwhile, MBAA acts as a covalent crosslinker to construct a polymeric network by linking acrylamide and/or LysMA. This covalent network can be formed either through direct crosslinking between alginate and PAAM chains or through the incorporation of alginate into a copolymer network consisting of acrylamide and/or LysMA. The resulting dual-crosslinked hydrogel structure combines ionic coordination and covalent bonding, which synergistically enhances mechanical strength, structural stability, and potentially the self-healing ability of the hydrogel.

### 3.2. Investigation of the Mechanical Properties of Composite Hydrogels

**Figure 2:**
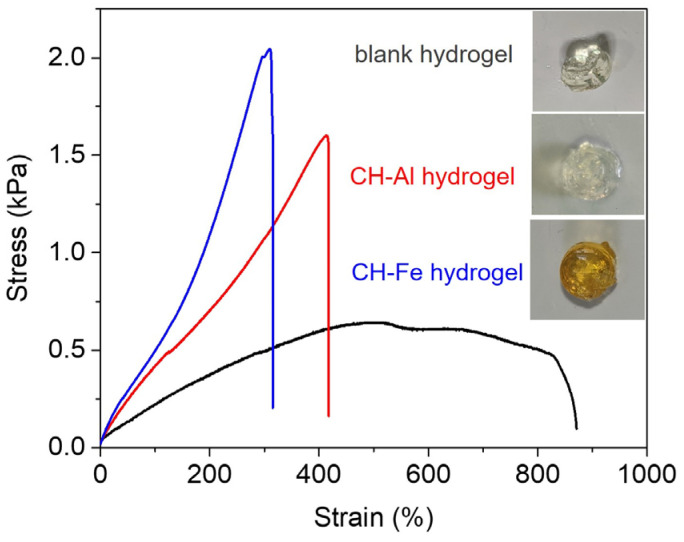
Tensile stress–strain curves of the blank hydrogel (black), CH-Al (red), and CH-Fe (blue). Inset: Optical images of the blank hydrogel (top), CH-Al (middle), and CH-Fe (bottom).

**Figure 3:**
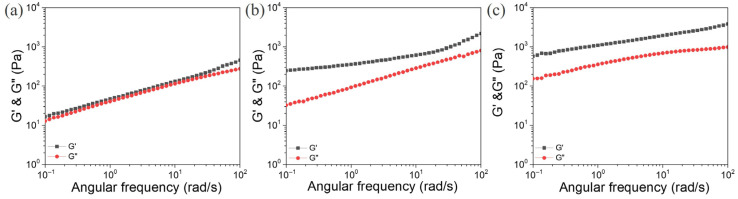
Rheological measurements of (**a**) blank hydrogel, (**b**) CH-Al, and (**c**) CH-Fe. G′ is shown in black; G″ is shown in red.

After successfully obtaining the blank hydrogel, CH-Al, and CH-Fe, we carried out tensile tests to evaluate and compare their mechanical properties. As shown in Figure 2 and summarized in Table 1, the incorporation of multivalent cations (Al^3+^ and Fe^3+^) significantly improved the tensile strength of the hydrogels compared to the blank hydrogel. Specifically, the tensile strengths of the blank hydrogel, CH-Al, and CH-Fe were measured at 0.49, 1.61, and 2.03 kPa, respectively, indicating a substantial enhancement in resistance to tensile failure upon ionic crosslinking. However, a trade-off was observed in terms of flexibility as the elongation at break decreased in the ionically crosslinked hydrogels. The blank hydrogel exhibited the highest elongation at break at 823.7%, while CH-Al and CH-Fe showed reduced values of 412.9% and 306.4%, respectively. This reduction in extensibility can be attributed to the more rigid and densely crosslinked network structure formed by the multivalent cations, which limits polymer chain mobility during deformation. Despite this, all three hydrogels demonstrated remarkable stretchability, with elongation ranging from 306.4% to 823.7%—well above the typical stretchability of human skin (60–75%)—highlighting their potential suitability for flexible and skin-mimicking biomedical applications. Compression tests were also performed on the hydrogels, revealing fracture stress values of 17.5 kPa for the blank hydrogel, 31.8 kPa for CH-Al, and 38.2 kPa for CH-Fe (Figure S1). Additionally, we investigated the rheological properties of the blank hydrogel, CH-Al, and CH-Fe using a rheometer (Figure 3 and Table 1). Figure 3 presents the storage modulus (G′) and loss modulus (G″) of the three hydrogels as functions of angular frequency (ω) within the linear viscoelastic region (0.1–100 rad/s). In all samples, G′ was consistently higher than G″, indicating dominant elastic behavior and confirming that the hydrogels primarily function as viscoelastic solids. Notably, the storage modulus, which reflects the stiffness and energy-storing capacity of the hydrogels, varied significantly among the samples. The blank hydrogel exhibited the lowest G′, suggesting a softer and more deformable structure, while CH-Al demonstrated a moderate increase in G′, and CH-Fe exhibited the highest value, indicating the greatest stiffness and structural integrity. Meanwhile, we examined the physical appearance of the hydrogels. The blank hydrogel exhibited poor structural integrity, while both CH-Al and CH-Fe demonstrated significantly improved formability and shape retention. Moreover, to gain deeper insight into the crosslinking degree of the blank hydrogel, CH-Al and CH-Fe, we conducted swelling behavior tests. As hydrogels with higher crosslinking density typically exhibit lower swelling ratios, this test serves as an indirect indicator of the extent of crosslinking [51]. We observed that CH-Fe exhibited the lowest swelling ratio, followed by CH-Al, while the blank hydrogel showed a significantly higher swelling ratio compared to both (Figure S2). These findings are consistent with the mechanical and rheological test results, further highlighting the reinforcing effect of ionic crosslinking with multivalent cations on the hydrogels’ mechanical and rheological performance.

### 3.3. Microstructural Analysis of Composite Hydrogels

**Figure 4:**
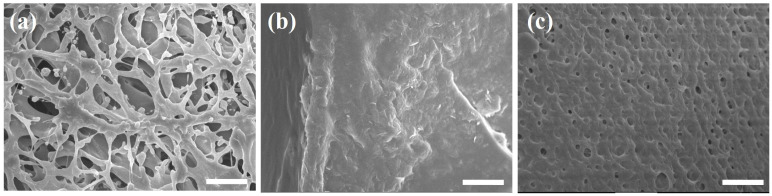
SEM images of (**a**) blank hydrogel, (**b**) CH-Al, and (**c**) CH-Fe. Scale bar: 10 µm.

Based on the tensile tests and rheological measurements, we observed a significant enhancement in the mechanical strength of the blank hydrogel following ionic exchange with multivalent cations. This mechanical reinforcement is primarily attributed to the formation of additional ionic crosslinks within the hydrogel matrix. Scanning electron microscope (SEM) analysis provided further insight into this phenomenon: while the blank hydrogel exhibited a distinct porous structure—indicative of a loosely packed polymer network—the CH-Al and CH-Fe hydrogels displayed a much denser, more compact morphology with minimal visible porosity. This structural transition can be explained by the interaction between the alginate chains and the multivalent cations (Al^3+^ and Fe^3+^), which led to the formation of a secondary, ionically crosslinked network superimposed on the existing covalent network formed by acrylamide polymerization. This denser internal structure contributes to the observed increase in tensile strength and storage modulus as seen in both the mechanical and rheological data. As shown in the SEM images of CH-Al and CH-Fe (Figure 4), a significant reduction in pore size and overall compact network morphology directly correlate with improved mechanical performance. However, this reinforcement comes at the cost of reduced chain mobility, which manifests as a decrease in the elongation at break. The more rigid network limits the polymer chains’ ability to stretch under tensile stress, thereby reducing flexibility and stretchability compared to the more porous and deformable blank hydrogel.

### 3.4. Testing of Composite Hydrogel Properties

**Figure 5:**
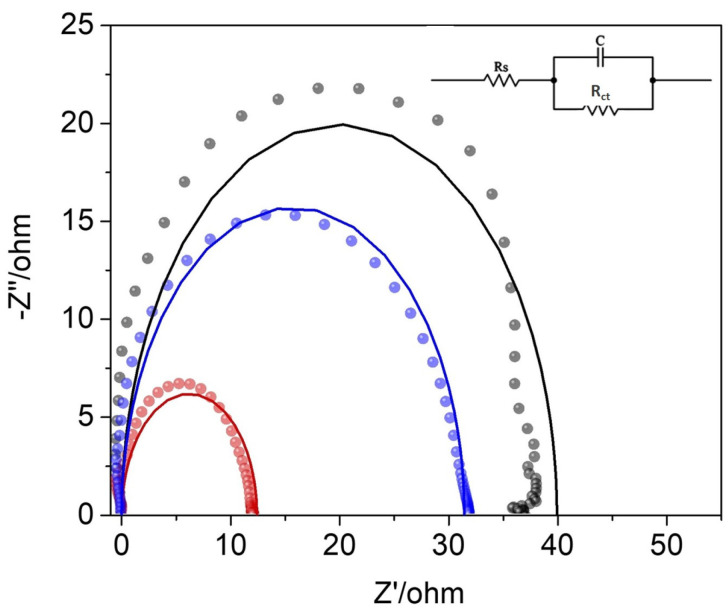
Nyquist impedance spectra of the blank hydrogel (black), CH-Al (red), and CH-Fe (blue). Inset: the equivalent circuit model consisting of ohmic resistance (Rs), charge transfer resistance (Rct), and capacitance (C).

To evaluate the conductive properties of the hydrogels, we employed electrochemical impedance spectroscopy (EIS) using an electrochemical workstation. As presented in Figure 5, the measured charge transfer resistance values for the blank hydrogel, CH-Al, and CH-Fe were 39.9, 12.4, and 31.4 ohms, respectively. These results clearly demonstrate that the incorporation of Al^3+^ ions significantly reduces the electrical resistance of the hydrogel. This reduction can be attributed to enhanced ionic mobility and the formation of a more compact, ionically crosslinked network that facilitates charge transport. Further details on the resistance, resistivity, and conductivity of the three hydrogels are provided in Table 2. Among the samples, CH-Al exhibited the highest electrical conductivity, highlighting the effectiveness of Al^3+^ in promoting ion transport within the hydrogel matrix [52]. This superior conductivity may be due to the smaller ionic radius and higher charge density of Al^3+^ compared to Fe^3+^, thereby improving the overall conductive pathway within the hydrogel [50,53]. In addition, we carried out biocompatibility evaluations of the CH-Al and CH-Fe hydrogels using the MTT assay (3-[4,5-dimethylthiazol-2-yl]-2,5-diphenyltetrazolium bromide) to assess their cytocompatibility with L929 fibroblast cells, with the aim of exploring their suitability for biomedical applications [54,55]. As shown in Figure S3, after 5 days of incubation, the cell viabilities reached 90% for CH-Al and 86% for CH-Fe, respectively. These results indicate that both hydrogels exhibit excellent biocompatibility, causing minimal cytotoxic effects.

**Figure 6:**
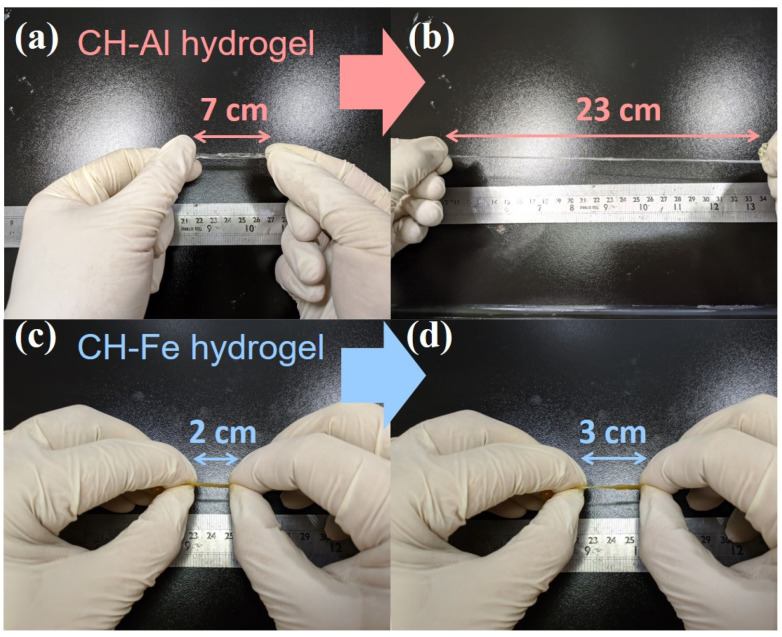
(**a**,**b**) Stretching tests of CH-Al. (**c**,**d**) Stretching tests of CH-Fe.

**Figure 7:**
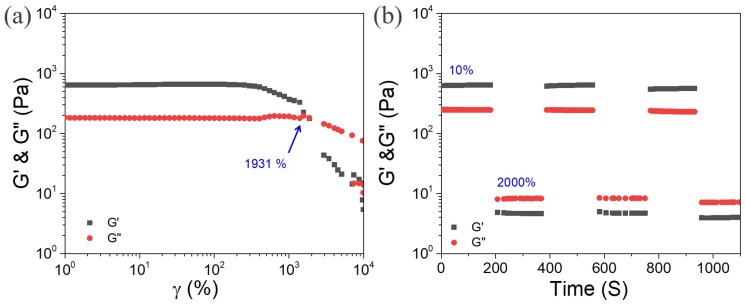
(**a**) Strain dependence of G′ (black) and G″ (red) for CH-Al. (**b**) Cyclic behavior of G′ (black) and G″ (red) of CH-Al under alternate-step strain sweep.

Next, we compared the stretchability of CH-Al and CH-Fe hydrogels. As shown in Figure 6, CH-Al was able to stretch up to 328.6% of its original length, while CH-Fe exhibited a maximum stretchability of only 150%. This stretchability trend closely mirrors the elongation at break observed in the tensile tests, reaffirming CH-Al’s superior flexibility. The greater stretchability of CH-Al can be attributed to the comparatively weaker ionic interactions between Al^3+^ ions and alginate chains, which facilitate more reversible deformation under mechanical stress. In contrast, Fe^3+^ ions form stronger coordination bonds with alginate, leading to a more rigid crosslinked network that limits chain mobility and thus reduces extensibility [56]. Following a comprehensive evaluation of the mechanical, electrical, and biological properties, CH-Al was identified as the optimal hydrogel for further investigation. We evaluated the self-healing efficiency of the CH-Al hydrogel through rheological recovery testing. As shown in Figure 7a, a strain amplitude sweep revealed that G′ and G″ intersected at a strain of 1931%, indicating the critical strain threshold at which the hydrogel transitions from a solid-like to a liquid-like state. This suggests that CH-Al maintains its structural integrity under relatively high deformation. To further examine its self-recovery behavior, we conducted an alternating step-strain test as illustrated in Figure 7b. A low strain of 10%—within the linear viscoelastic region—was used to monitor the hydrogel’s baseline mechanical response. To simulate mechanical damage, a high strain of 2000%, which exceeds the critical threshold, was applied to disrupt the internal crosslinked network. Under this large deformation, G′ dropped sharply and became smaller than G″, signifying a transition to a flowable state due to network breakdown. Remarkably, once the strain was reduced back to 10%, G′ rapidly recovered to nearly its initial value, demonstrating the hydrogel’s excellent self-healing performance (Table S1). We also confirmed the hydrogel’s outstanding self-healing ability by injecting it through a syringe (Figure S4). This ability to recover its mechanical properties after extreme deformation is attributed to the reversible ionic interactions between alginate chains and Al^3+^ ions. Such dynamic crosslinking not only reinforces the hydrogel’s mechanical strength but also enables structural reformation after disruption, making CH-Al a promising candidate for applications requiring durability and resilience under stress. Finally, adhesion tests were performed on CH-Al using a variety of substrates, including glass, plastic, steel weights, paper, rubber, wood, and aluminum. As illustrated in Figure S5, CH-Al demonstrated strong adhesion across all tested materials, likely attributed to the presence of LysMA, whose primary amine and methacrylate groups facilitate both covalent and non-covalent interactions with the substrates [57].

## 4. Conclusions

In this study, we developed a series of conductive hydrogels by integrating FeCl_3_ and AlCl_3_ into an alginate/polyacrylamide matrix, resulting in CH-Fe and CH-Al, respectively. We conducted a comprehensive evaluation of how these multivalent metal ions influence the hydrogels’ mechanical strength and electrical conductivity. Among the formulations, CH-Al emerged as the most effective, exhibiting a remarkable 329% increase in tensile strength and a 323% boost in conductivity relative to the unmodified hydrogel (blank hydrogel). Notably, CH-Al also demonstrated near-complete self-healing efficiency, with a recovery rate approaching 100%. The superior performance of CH-Al is attributed to the presence of Al^3+^ ions, which not only enhance structural reinforcement through secondary crosslinking but also promote the formation of dynamic, conductive pathways via coordination interactions with the polymer chains. These reversible metal–ligand interactions underpin the hydrogel’s autonomous self-healing behavior, enabling it to rapidly recover its physical integrity and functional properties following mechanical disruption. Altogether, the CH-Al hydrogel offers an integrated platform combining high conductivity, mechanical robustness, and efficient self-healing, making it a promising candidate for next-generation bioelectronics and wearable sensors.

## Figures and Tables

**Table 1:** Mechanical properties of composite hydrogels (n = 3).

Hydrogel	Tensile Strength (kPa)	Elongation at Break (%)	G′ (Pa)	G″ (Pa)
Blank	0.49 ± 0.02	823.7 ± 2.9	123.5 ± 2.8	95.8 ± 1.7
CH-Al	1.61 ± 0.04	412.9 ± 3.4	642.7 ± 4.5	248.1 ± 2.0
CH-Fe	2.03 ± 0.06	306.4 ± 2.1	1681.6 ± 10.2	534.7 ± 3.1

**Table 2:** Electrical properties of the composite hydrogels (n = 3).

Hydrogel	Rct (Ω)	Resistivity (Ω Cm)	Conductivity (μS/cm)
Blank	39.91 ± 3.21	4989 ± 85	200.4 ± 12.9
CH-Al	12.40 ± 1.02	1550 ± 26	645.2 ± 31.0
CH-Fe	31.42 ± 5.10	3927 ± 102	254.7 ± 10.8

## Data Availability

The original contributions presented in this study are included in the article/Supplementary Material. Further inquiries can be directed to the corresponding authors.

## Supplementary Materials

The following supporting information can be downloaded at https://www.mdpi.com/article/10.3390/ma18163871/s1, Figure S1: Compressive stress–strain curves. Figure S2: The swelling ratio of hydrogels. Figure S3: Cell viability. Table S1: Summary of G′ and G″ values of CH-Al under alternate-step strain sweep. Figure S4: Self-healing test. Figure S5: Adhesion images.

## Abbreviations

The following abbreviations are used in this manuscript:

PAAM	Polyacrylamide
CH	Composite hydrogels
AAM	Acrylamide
LysMA	Methacrylated lysine
MBAA	*N*,*N*′-methylenebisacrylamide
G′	Storage modulus
G″	Loss modulus
SEM	Scanning electron microscope
EIS	Electrochemical impedance spectroscopy
