# Supplementary material for: Effect of Metal Ions on the Conductivity, Self-Healing, and Mechanical Properties of Alginate/Polyacrylamide Hydrogels
Source: Materials (Basel). 2025 Aug 18;18(16):3871. doi: 10.3390/ma18163871 (PMC12387799; doi:10.3390/ma18163871)
Supplement: Supplementary file 1 [file materials-18-03871-s001.zip › materials-3674278-supplementary.pdf]

materials

*Article*

# Effect of Metal Ions on the Conductivity, Self-Healing, and Mechanical Properties of Alginate/Polyacrylamide Hydrogels

**Chen-Kang Chen [1], Chien-Yin Lin [1], Rajan Deepan Chakravarthy [2], Yu-Hsu Chen [3], Chieh-Yi Chen [4,*], Hsin-Chieh Lin [2,5,*] and Mei-Yu Yeh [1,*]**

1 Department of Chemistry, Chung Yuan Christian University, Taoyuan City 320314, Taiwan; za14253698a@gmail.com (C.-K.C.); lin.sylvia99999@gmail.com (C.-Y.L.)
2 Department of Materials Science and Engineering, National Yang Ming Chiao Tung University, Hsinchu 300093, Taiwan; deepannycu@gmail.com
3 Department of Orthopedic Surgery, Taoyuan General Hospital, Ministry of Health and Welfare, Taoyuan 330215, Taiwan; magister.yuhsu@gmail.com
4 Neurosurgical Department, Taoyuan General Hospital, Ministry of Health and Welfare, Taoyuan 330215, Taiwan
5 Center for Intelligent Drug Systems and Smart Bio-Devices (IDS[2]B), National Yang Ming Chiao Tung University, Hsinchu 30068, Taiwan
* Correspondence: blacatae@gmail.com (C.-Y.C.); hclin45@nycu.edu.tw (H.-C.L.); myyeh@cycu.edu.tw (M.-Y.Y.)

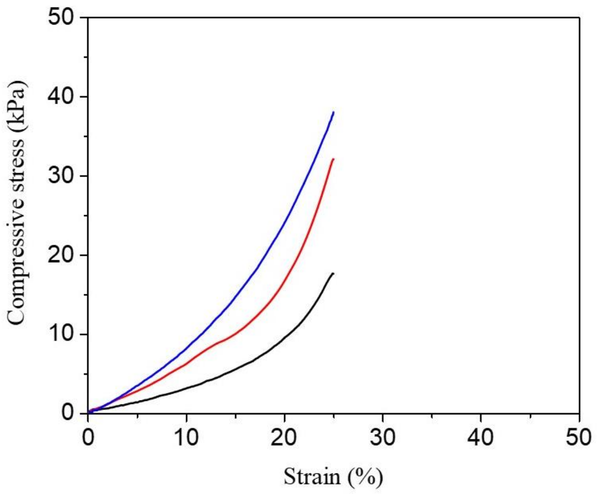


**Figure S1.** Compressive stress–strain curves of the blank hydrogel (black), CH-Al hydrogel (red), and CH-Fe hydrogel (blue).

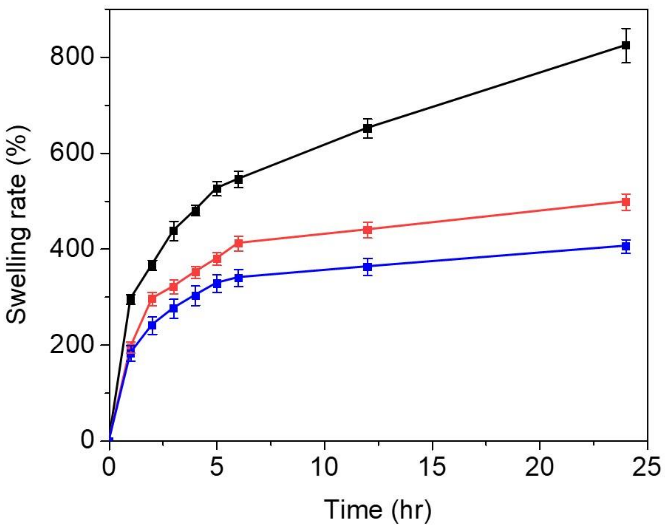


**Figure S2.** The Swelling ratio of the blank hydrogel (black), CH-Al hydrogel (red), and CH-Fe hydrogel (blue) (n=3).

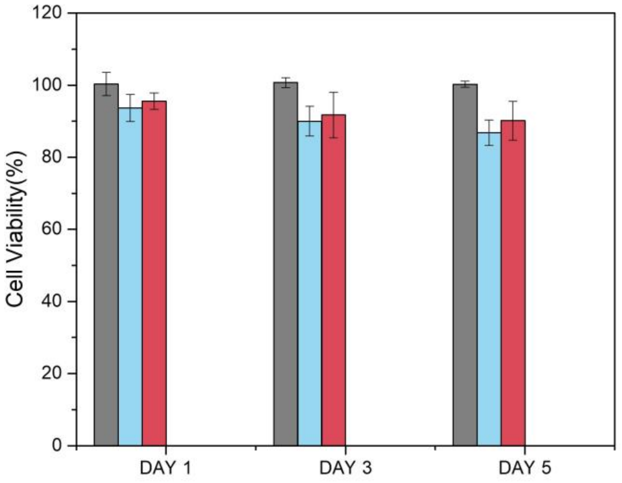


**Figure S3.** Cell viability of L929 cells cultured on the blank hydrogel (black), CH-Al hydrogel (red), and CH-Fe hydrogel (blue) after 1, 3, and 5 days (n=3).

**Table S1.** Summary of G′ and G″ values of CH-Al under alternate-step strain sweep.

| Step | γ= 10%<br>G′ & G″ (Pa) | γ= 2000%<br>G′ & G″ (Pa) | % recovery (γ=10%) |
|---|---|---|---|
| 1 | 637.6, 247.8 | 4.7, 8.2 | / |
| 2 | 634.3, 245.8 | 4.7, 8.3 | 99.5 |
| 3 | 575.8, 233.5 | 4.0, 7.2 | 90.3 |

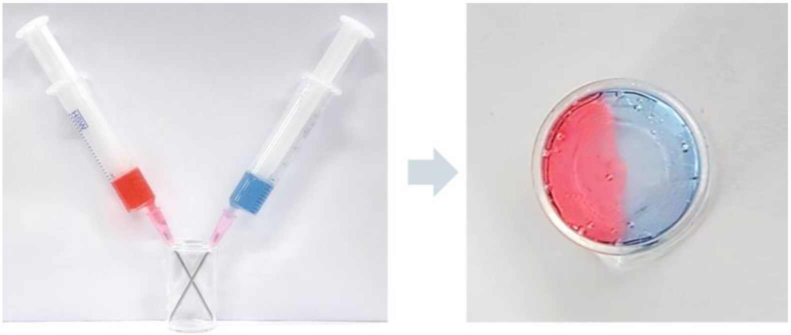

**Figure S4.** Self-healing test of CH-Al. CH-Al was separately dyed blue and pink, and its healing behavior was observed through syringe injection.

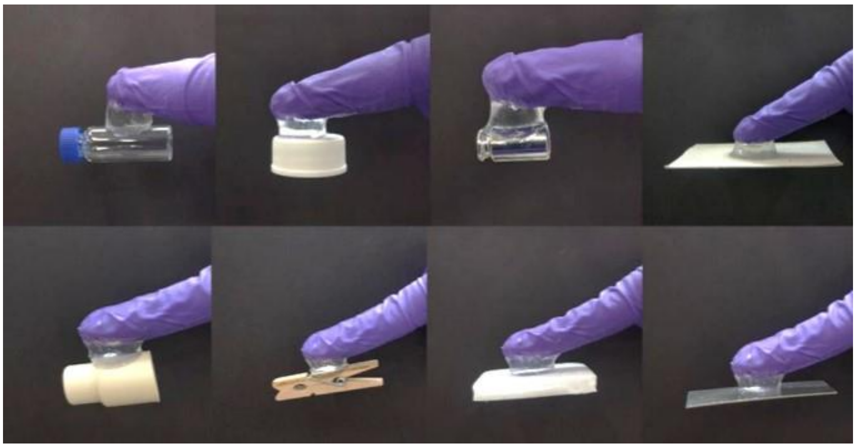

**Figure S5.** Adhesion images of CH-Al on various substrates, including glass, plastic, steel weights, paper, rubber, wood, and aluminum.
